# Editorial—2026 TPJ Fellows

**DOI:** 10.1111/tpj.71164

**Published:** 2026-10-07

**Authors:** Zhongchi Lui, Magda Julkowska, Alisdair R. Fernie, Katherine Denby

*The Plant Journal* is delighted to announce the successful 2026 TPJ Fellows—**Martina Legris, Adam Bentham** and **Furong Liu**. These early career group leaders have exciting scientific programmes and impressive research track records, as well as contributions to the wider plant science community.

**Martina** is from Argentina. She completed her Bachelor's, Master's and PhD studies in biology at the University of Buenos Aires, doing research at the Leloir Institute. From early in her career, she has been fascinated by plant developmental plasticity, and since her Master's degree, has been working on plant development in response to light and temperature signals from the environment. During her PhD, under the supervision of Jorge Casal, she focused on the interaction between light and temperature signalling and found that the activity of the light receptor phytochrome B is also regulated by temperature. For her postdoc, she moved to Christian Fankhauser's laboratory in Lausanne, Switzerland, where she focused on the blue light receptors phototropins. There she described mechanisms of directional signal perception in leaves and hypocotyls. Since September 2024, she has been an Assistant Professor at the University of Neuchâtel. Her team studies light and temperature effects on shoot meristems and the consequent changes in shoot architecture. In addition to doing research, Martina enjoys teaching plant biology and organizing science outreach activities.

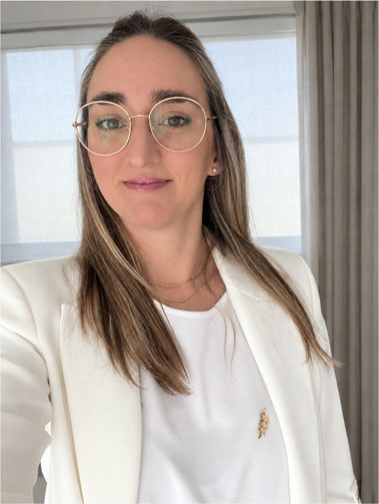

**Adam** is an Assistant Professor in the Department of Biosciences at Durham University and Co‐Director of the Centre for Programmable Biological Matter. His research combines structural biology, AI‐based protein design and plant synthetic biology to study and engineer plant–microbe interactions, with a focus on plant intracellular immune receptors (NLRs) and their recognition of pathogen effector proteins for the rational design of disease resistance. Adam holds a Bachelor of Biotechnology (Hons) from Flinders University, South Australia and a PhD in structural biology from the University of Queensland, where he worked in the group of Prof. Boštjan Kobe. In 2017, he joined Prof. Mark Banfield's group at the John Innes Centre as a postdoctoral researcher, where he worked on determining structures of pathogen effectors in complex with their host targets and began engineering plant immune receptors to expand disease resistance in staple crops. In 2023, he moved to the laboratory of Prof. Nick Talbot at The Sainsbury Laboratory investigating the function of effector proteins from *Magnaporthe oryzae* through computational structure prediction and design, before his appointment to his current position in 2024.

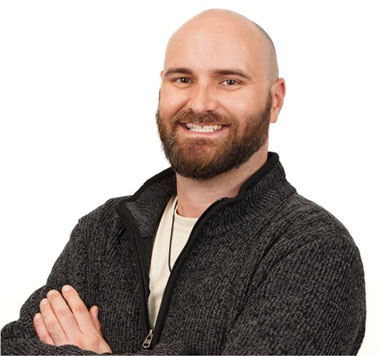

**Furong** is a Principal Investigator in the School of Advanced Agricultural Sciences and the State Key Laboratory of Gene Function and Modulation Research at Peking University. He received his B.S. and M.S. degrees from Northwest A&F University and earned his Ph.D. in Plant Pathology from the University of California, Davis. He subsequently conducted postdoctoral research at UC Berkeley, where he was affiliated with the Innovative Genomics Institute (IGI), before joining Peking University in 2025. As a molecular plant pathologist, he has contributed to elucidating the structural and functional mechanisms of plant immune receptors, including the assembly and activation of immune complexes and downstream defence signalling. His team integrates structural biology, genetics, cell biology and molecular interaction profiling to define key regulatory nodes in plant immune networks. His current research centres on plant–pathogen interactions and disease resistance in rice and wheat, with a focus on harnessing genome‐editing technologies to broaden pathogen recognition and precisely engineer broad‐spectrum resistance in major crops.

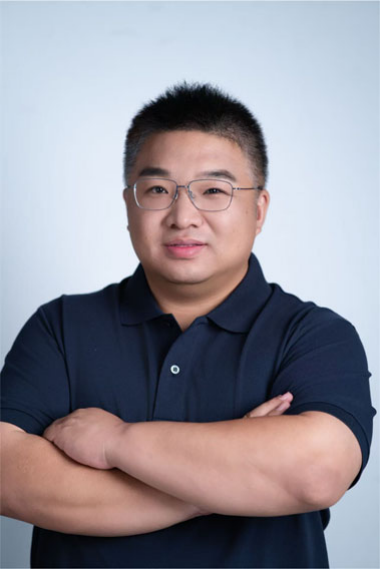

Congratulations to Martina, Adam and Furong. We welcome them to The Plant Journal team and look forward to supporting their careers.

